# Effects of nutritional conditions on growth and biochemical composition of *Tetraselmis* sp.

**DOI:** 10.1186/s12944-016-0378-1

**Published:** 2017-02-20

**Authors:** Mouna Dammak, Bilel Hadrich, Ramzi Miladi, Mohamed Barkallah, Faiez Hentati, Ridha Hachicha, Céline Laroche, Philippe Michaud, Imen Fendri, Slim Abdelkafi

**Affiliations:** 1 Unité de Biotechnologie des Algues, Biological Engineering Department, National School of Engineers of Sfax, University of Sfax, Sfax, Tunisia; 2 Université Clermont Auvergne, Université Blaise Pascal, Institut Pascal, BP 10448, F-63000 Clermont-Ferrand, France; 3 CNRS, UMR 6602, IP, F-63178 Aubière, France; 4 Laboratory of Plant Biotechnology, Faculty of Sciences of Sfax, University of Sfax, Sfax, Tunisia

**Keywords:** Microalgae, *Tetraselmis* sp., Starch, Biomass, Bioactive compounds, Box-Behnken Design

## Abstract

**Background:**

This study aimed to maximize biomass concentration, biomass productivity and biochemical composition of the marine microalga *Tetraselmis* sp.

**Methods:**

In the current study, Box-Behnken Design was used to model the effect of NaNO_3_, NaH_2_PO_4_, metals and vitamins in the F/2 medium on the growth, total chlorophylls, carotenoids and starch contents. The total chlorophylls content was quantified by spectrophotometry. The FT-IR spectroscopy was used to estimate the biochemical compositions of *Tetraselmis* sp. grown under both optimized medium culture for starch production and standard culture medium.

**Results:**

Finalized NaNO_3_ (1.76 mM), NaH_2_PO_4_ (0.018 mM), metals (1500 μL.L^−1^) and vitamins (312.5 μL.L^−1^) concentrations, generated an increase in biomass concentration up to 5.72 g.L^−1^ which contributed to an increase about 2.4-fold than that of the standard conditions of biomass productivity (408.57 mg.L^−1^.day^−1^). The maximum value of carotenoids content (0.3 mg.g DW^−1^) was achieved at the highest level of all factors. The total chlorophylls content reached also its maximum (5.18 mg.g DW^−1^) at high nitrate (1.76 mM), phosphate (0.054 mM), metals and vitamins concentrations, while the maximum starch content (42% DW) was achieved with low nitrate and phosphate concentrations (0.58 mM and 0.027 mM) and with metals and vitamins limitations. Thus, the nitrogen, phosphorus, metals and vitamins limitations led to divert the metabolism for the starch biosynthesis.

**Conclusions:**

The high biomass concentration productivity and starch production make *Tetraselmis* sp. strain a good candidate for biotechnological applications.

## Background

The rising crude oil prices, scarcity of fossil fuels, increasing environmental pollution due to CO_2_ and NO_x_ emissions and increasing energy demand have led to look for renewable energy sources such as bioethanol, biodiesel and biohydrogen [1]. In this context, microalgae were considered as one of the important sources of biofuels enjoying the advantage of accumulation of lipids (20–50% DW) and other compounds such as carbohydrates, pigments, proteins and antioxidants. Microalgae provide carbohydrates (exopolysaccharides, cell wall polysaccharides and starch) which can be used for fermentation by yeast, bacteria or fungi. For instance, *Tetraselmis* sp. has been suggested as a good candidate for bioethanol production owing to its high biomass and starch production [2, 3]. Nutrients disponibility in microalgal culture can regulate their growth and biochemical composition [4]. However, biomass and macromolecules (pigments and starch) production depends not only on primary nutrients (nitrogen and phosphorus) but also on metals (iron, zinc, copper, manganese, molybdenum, cobalt), some ions such as Cl^−^, Ca^2+^, Na^2+^, SO_4_^2−^ and vitamins. These micronutrients are needed for electron transport in photosynthesis and cellular respiration, sulphate and nitrate reductions [5]. Furthermore, nutrients limitation essentially phosphorus (P) and nitrogen (N) reduces cells growth and protein synthesis and increases starch or lipid production [4]. For example, *T. subcordiformis* was revealed as a great starch producing green microalga under light limitation (50 μmol.m^−2^.s^−1^) and nitrogen deprivation [2]. In addition, the limitation of macroelements such as nitrogen, phosphorus and sulphide can also induce starch accumulation [5–7]. Recently, Markou et al. [8] have reported that phosphorus limitation leads to the accumulation of carbohydrates and lipids. In the current study, the green microalga *Tetraselmis* sp. isolated from Tunisian seawater, was investigated to produce biomass, chlorophylls, carotenoids and starch. No special attention has been given yet to optimize the photoautotrophic culture of this strain for biomass, production and metabolites biosynthesis using the Response Surface Methodology (RSM). The photosynthetic performance of *Tetraselmis* sp. evaluated by total chlorophylls biosynthesis, was simultaneously optimized with the starch content. Therefore, the improvement of cultivation factors can reduce the cost of microalgae culture by reducing their nutrients consumption and maximizing their biomass and starch productions. Box-Behnken Design (BBD) is a useful methodology for testing the effects of the important nutritional components as their interactions would maximize cell growth, and metabolites synthesis by *Tetraselmis* sp. under photoautotrophic cultivation. Thus, the relationship of these products was studied under different conditions.

## Methods

### Strain and culture conditions

The green microalga, *Tetraselmis* sp. (V_2_), was isolated from the Gulf of Gabes (Mediterranean Sea) along the coast of Sidi Mansour (Tunisia) using different cell isolation techniques. It was identified relying on its morphological and phylogenetic analysis (23S rDNA). Microscopic observation showed a motile, bilaterally symmetrical cells of 7–20 μm in bredth, 10–25 μm in length and 6.5–18 μm in thickness [9]. It was characterized by their chloroplasts dorsoventrally lobed, yellow-green color and usually with pyrenoid containing many starch grains [9]. *Tetraselmis* sp. was cultivated at 25 ± 1 °C under continuous light conditions (84 μmol.m^−2^.s^−1^) with white fluorescent lamps (Compact Fluorescent Lamp, Superlight, Tunisia) and at pH 7. Experimental cultures (150 mL) were inoculated with 10% of mother culture and incubated for 15 days. The microalgae was cultivated in natural seawater with the following F/2 nutrients (per litre) [10]: 1 mL NaNO_3_ (75 g.L^−1^), 1 mL NaH_2_PO_4_ (5 g.L^−1^), 1 mL trace metal solution and 0.5 mL vitamins solution. Trace metal solution was prepared in pure water containing (per liter): 3.15 g FeCl_3_,6H_2_O; 4.36 g Na_2_EDTA,2H_2_O; 1 mL CuSO_4_,5H_2_O (9.8 g.L^−1^); 1 mL Na_2_MoO_4_,2H_2_O (6.3 g.L^−1^); 1 mL ZnSO_4_,7H_2_O (22 g.L^−1^); 1 mL CoCl_2_,6H_2_O (10 g.L^−1^); 1 mL MnCl_2_,4H_2_O (180 g.L^−1^). The vitamin solution was prepared in pure water containing (per liter): 200 mg thiamine HCl (vitamin B1), 1 mL biotin (vitamin H) (1 g.L^−1^) and 1 mL cyanocobalamin (vitamin B12) (1 g.L^−1^). In optimization experiments, the algal cells were cultivated at three levels of four F/2 nutrients. Extracellular concentrations of NaNO_3_ and NaH_2_PO_4_ are shown in Table 1. Extracellular concentrations of metals and vitamin solution components at three levels (−1, 0, +1) are displayed in Table 2.

**Table 1:** Variables and experimental levels for F/2 medium culture optimization

Factors	Symbol	Levels		
		−1	0	+1
NaNO_3_ (mM)	*x*_1_	0.58	1.17	1.76
NaH_2_PO_4_ (mM)	*x*_2_	0.018	0.036	0.054
Trace metals (μL.L^-1^)	*x*_3_	500	1000	1500
Vitamins (μL.L^-1^)	*x*_4_	250	375	500

**Table 2:** Extracellular metals and vitamins concentrations

Coded Levels	−1	0	+1
Extracellular metals concentration			
FeCl_3_,6H_2_O (M)	0.5 10^−5^	1 10^−5^	1.5 10^−5^
Na_2_EDTA,2H_2_O (M)	0.5 10^−5^	1 10^−5^	1.5 10^−5^
CuSO_4_,5H_2_O (M)	2 10^−8^	4 10^−8^	6 10^−8^
Na_2_MoO_4_,2H_2_O (M)	1.5 10^−8^	3 10^−8^	4.5 10^−8^
ZnSO_4_,7H_2_O (M)	4 10^−8^	8 10^−8^	12 10^−8^
CoCl_2_,6H_2_O (M)	2.5 10^−8^	5 10^−8^	7.5 10^−8^
MnCl_2_,4H_2_O (M)	4.5 10^−7^	9 10^−7^	13.5 10^−7^
Extracellular vitamins concentration			
Thiamine HCl (vitamin B1) (M)	1.48 10^−7^	2.22 10^−7^	2.96 10^−7^
Biotin (vitamin H) (M)	1.025 10^−9^	1.5375 10^−9^	2.05 10^−9^
Cyanocobalamin (vitamin B12) (M)	1.845 10^−10^	2.7675 10^−10^	3.69 10^−10^

### Growth measurement

The microalgae growth was determined by estimating cells concentration. After cultivation, cells were harvested by centrifugation at 5000 × g for 10 min at the late log phase. Pellets were dried at 105 °C until their weight became constant (DW).

### Pigments and starch contents determination

To quantify pigments, 1 mL of culture was centrifuged at 5000 × g for 10 min. The pellet was suspended in 1 mL ethanol and sonicated at 65 °C for 30 min. After sonication, the solution was centrifuged at 5000 × g for 5 min and A_666_, A_653_, and A_470_ were measured to quantify pigments using the equations (1), (2), (3) and (4) as described previously [11, 12];

1 $$ \left[\mathrm{Chlorophyll}\ \mathrm{a}\right]\ \left(\mathrm{mg}.{\mathrm{L}}^{-1}\right)=15.65\times {A}_{666}\hbox{--} 7.340 \times {A}_{653} $$

2 $$ \left[\mathrm{Chlorophyll}\ \mathrm{b}\right]\ \left(\mathrm{mg}.{\mathrm{L}}^{-1}\right) = 27.05 \times {A}_{653}\hbox{--}\ 11.21 \times {A}_{666} $$

3 $$ \left[\mathrm{Total}\ \mathrm{Chlorophylls}\right]\ \left(\mathrm{mg}.{\mathrm{L}}^{-1}\right) = \left[\mathrm{Chlorophyll},, \mathrm{a}\right] + \left[\mathrm{Chlorophyll},, \mathrm{b}\right] $$

4 $$ \left[\mathrm{Carotenoids}\right]\ \left(\mathrm{mg}.{\mathrm{L}}^{-1}\right)=\left(1000 \times {A}_{470}\hbox{--}\ 2.860 \times \left[\mathrm{Chlorophyll}\ \mathrm{a}\right]\hbox{--}\ 85.9 \times \left[\mathrm{Chlorophyll}\ \mathrm{b}\right]\right)/\ 245 $$

Starch content in pellet was determined as described by Hirst et al. [13] and Xiao et al. [14] using the equation (5):

5 $$ \mathrm{Starch}\ \mathrm{content}\ \left(\mathrm{g}.{\mathrm{L}}^{-1}\right) = \frac{A_{600}}{2.294} $$

### Experimental design and data analysis

The effects of F/2 medium components on cells growth, pigments and starch productions by *Tetraselmis* sp. were evaluated and analyzed by Box-Behnken methodology [15].

The experiment design contained 27 trials (Table 3). Table 1 shows the four independent variables which are extracellular NaNO_3_ concentration (*x*_1_), extracellular NaH_2_PO_4_ concentration (*x*_2_), the metal solution initial volume (*x*_3_) and the vitamin solution initial volume (*x*_4_). These factors were studied at three levels, low (−1), medium (0) and high (+1).

**Table 3:** Experimental results of Box-Behnken design

Run N^o^	Factors				Responses			
	NaNO_3_ (mM)	NaH_2_PO_4_ (mM)	Metals (μL.L^−1^)	Vitamins (μL.L^−1^)	Biomass (g.L^−1^)	Total chlorophylls content (mg.g DW^−1^)	Carotenoids content (mg.g DW^−1^)	Starch content (g.g DW^−1^)
1	0.58	0.018	1000	375	2.2	0.72	0.13	0.38
2	1.76	0.018	1000	375	3.28	2.5	0.08	0.12
3	0.58	0.054	1000	375	1.19	3.7	0.2	0.25
4	1.76	0.054	1000	375	1.7	4.62	0.28	0.3
5	1.17	0.036	500	250	3.37	2.04	0.06	0.38
6	1.17	0.036	1500	250	4.57	1.15	0.12	0.26
7	1.17	0.036	500	500	3.14	3.32	0.15	0.32
8	1.17	0.036	1500	500	2.15	3.69	0.16	0.32
9	1.17	0.036	1000	375	2.6	3.59	0.17	0.32
10	0.58	0.036	1000	250	1.95	1.41	0.17	0.42
11	1.76	0.036	1000	250	2.91	2.5	0.18	0.23
12	0.58	0.036	1000	500	1.77	2.5	0.24	0.29
13	1.76	0.036	1000	500	2.1	4.5	0.23	0.33
14	1.17	0.018	500	375	3.39	1.7	0.09	0.29
15	1.17	0.054	500	375	2.61	3.48	0.1	0.33
16	1.17	0.018	1500	375	4.8	1.3	0.05	0.36
17	1.17	0.054	1500	375	1.72	3.5	0.26	0.3
18	1.17	0.036	1000	375	2.37	3.71	0.17	0.33
19	0.58	0.036	500	375	1.94	2.3	0.16	0.37
20	1.76	0.036	500	375	1.62	3.7	0.21	0.31
21	0.58	0.036	1500	375	1.85	2	0.18	0.33
22	1.76	0.036	1500	375	2.55	3.61	0.2	0.25
23	1.17	0.018	1000	250	5.2	0.8	0.04	0.28
24	1.17	0.054	1000	250	2.1	3.38	0.19	0.38
25	1.17	0.018	1000	500	1.99	2.42	0.16	0.32
26	1.17	0.054	1000	500	2.2	4.92	0.23	0.28
27	1.17	0.036	1000	375	2.15	3.72	0.19	0.37

The biomass concentration (*Y*_1_), total chlorophylls (*Y*_2_), carotenoids (*Y*_3_) and starch (*Y*_4_) contents were analyzed as asked responses of the experiments design.

Experimental data were fitted with a second order polynomial model (Eq. 6):

6 $$ \widehat{Y}={\beta}_0+\sum {\beta}_{\mathrm{i}}{x}_{\mathrm{i}}+\sum {\beta}_{\mathrm{i}\mathrm{i}}{x_{\mathrm{i}}}^2+\sum {\beta}_{\mathrm{i}\mathrm{j}}{x}_{\mathrm{i}}{x}_{\mathrm{j}} $$

Where *Ŷ*: response variable; β_0_ : constant coefficient; *x*_i_ and *x*_j_: uncoded variables ranging between the minimum and the maximum concentrations of the different factors; β_i_, β_ii_ and β_ij_: coefficients for the linear, quadratic, and interaction effects, respectively.

After *Tetraselmis* sp. cultivation under different nutritional conditions, the statistical analysis of Box-Behnken Design of experimental results was carried out using STATISTICA software 8.0 (Stat Soft. Inc 2008).

The RSM is an efficient statistical tool to investigate the factors effect and interactions with a minimum number of experiments [16, 17]. Thus, to optimize the biomass, total chlorophylls, carotenoids and starch production (*Y*_1_, *Y*_2_, *Y*_3_, *Y*_4_) by *Tetraselmis* sp., a total of 27 experiments were performed in this study (Table 3). Accordingly, *P*-values were carried out for regression analysis and to evaluate the significance of factors’ effects of the first (*x*_1_, *x*_2_, *x*_3_, *x*_4_) and the second (*x*_1_^2^, *x*_2_^2^, *x*_3_^2^, *x*_4_^2^) order polynomials, and their interactions (*x*_1_ *x*_2_, *x*_1_ *x*_3_, *x*_1_ *x*_4_, *x*_2_ *x*_3_, *x*_2_ *x*_4_, *x*_3_ *x*_4_).

The optimum responses were obtained with the “Response Desirability Profiling” tool of STATISTICA Software.

### FT-IR spectroscopy

The absorption spectra of the samples were obtained using FT-IR spectroscopy (Agilent Technologies Spectrophotometer, Cory 630 FT-IR). The dried cells obtained from 5 mL of culture were pulverized to powder and pressed into tablet.

Spectrum software was employed to process the FT-IR spectra. The transmittance spectra were measured between 600 and 4000 cm^-1^ using 10 scans and 4 cm^-1^ resolution.

## Results

### Optimization of F/2 medium components for biomass, total chlorophylls, carotenoids and starch productions by *Tetraselmis* sp. strain V_2_

#### Combined effects of modified-F/2 medium components on *Tetraselmis* sp. growth

Using the multiple regression analysis, the growth response (*Y*_1_) second-order equation was estimated as below to explain the *Tetraselmis* sp. biomass production (Eq. 7):

7 $$ {\widehat{Y}}_1=7.52\ \hbox{--} 1.86{x_1}^2\hbox{--} 160.45\ {x}_2 + 1.73\ 1{0}^{-6}{x_3}^2\hbox{--} 2.6\ 1{0}^{-2}{x}_4\hbox{--} 6.4\ 1{0}^{-2}{x}_2{x}_3 + 0.36\ {x}_2{x}_4\hbox{--} 8.80\ 1{0}^{-6}{x}_3{x}_4\left( P-\mathrm{value} < 0.05\right) $$

The analysis of variance (ANOVA) was applied to check the significance of the second-order polynomial equation (Eq. 7) by fitting the experimental data shown in Table 4. The *P*-values were used to test the variables significance. As seen in Table 4, the determination coefficient (R^2^ = 0.94) highlighted the significance of the model and the lack of fit value is not significant (*P*-value = 0.29 ˃ 0.05).

**Table 4:** Analysis of variance of the model for biomass production

Source	Sum of squares	df	Mean square	*F* value	*P* value
*x*_1_	0.88	1	0.88	17.49	0.05
*x*_2_	4.01	1	4.01	79.37	<0.05
*x*_3_	0.20	1	0.20	4.05	0.18
*x*_4_	3.79	1	3.79	74.98	<0.05
*x*_1_ *x*_2_	0.08	1	0.08	1.60	0.33
*x*_1_ *x*_3_	0.26	1	0.26	5.13	0.15
*x*_1_ *x*_4_	0.09	1	0.09	1.95	0.29
*x*_2_ *x*_3_	1.32	1	1.32	26.11	<0.05
*x*_2_ *x*_4_	2.73	1	2.73	54.09	<0.05
*x*_3_ *x*_4_	1.19	1	1.19	23.68	<0.05
*x*_1_^2^	2.25	1	2.25	44.50	<0.05
*x*_2_^2^	0.37	1	0.37	7.39	0.11
*x*_3_^2^	0.99	1	0.99	19.58	<0.05
*x*_4_^2^	0.84	1	0.84	16.74	0.05
Lack of fit	1.42	10	0.14	2.81	0.29
Pure error	0.10	2	0.05		
Total	25.82	26			

R^2^ = 0.94

The lower *P*-values correspond to the high significance of the variables. In this study, *P*-values for *x*_1_^2^, *x*_2_, *x*_3_^2^, *x*_4_, *x*_2_ *x*_3_, *x*_2_ *x*_4_ and *x*_3_ *x*_4_ are less than 0.05, which explains the significance of model terms.

This indicates the strong effect of all of F/2 nutrients on *Tetraselmis* sp. strain V_2_ growth. In addition, the negative regression coefficient of NaH_2_PO_4_ (*x*_2_) and vitamins (*x*_4_) suggested an antagonist effect of these two components on biomass production.

**Fig. 1:**
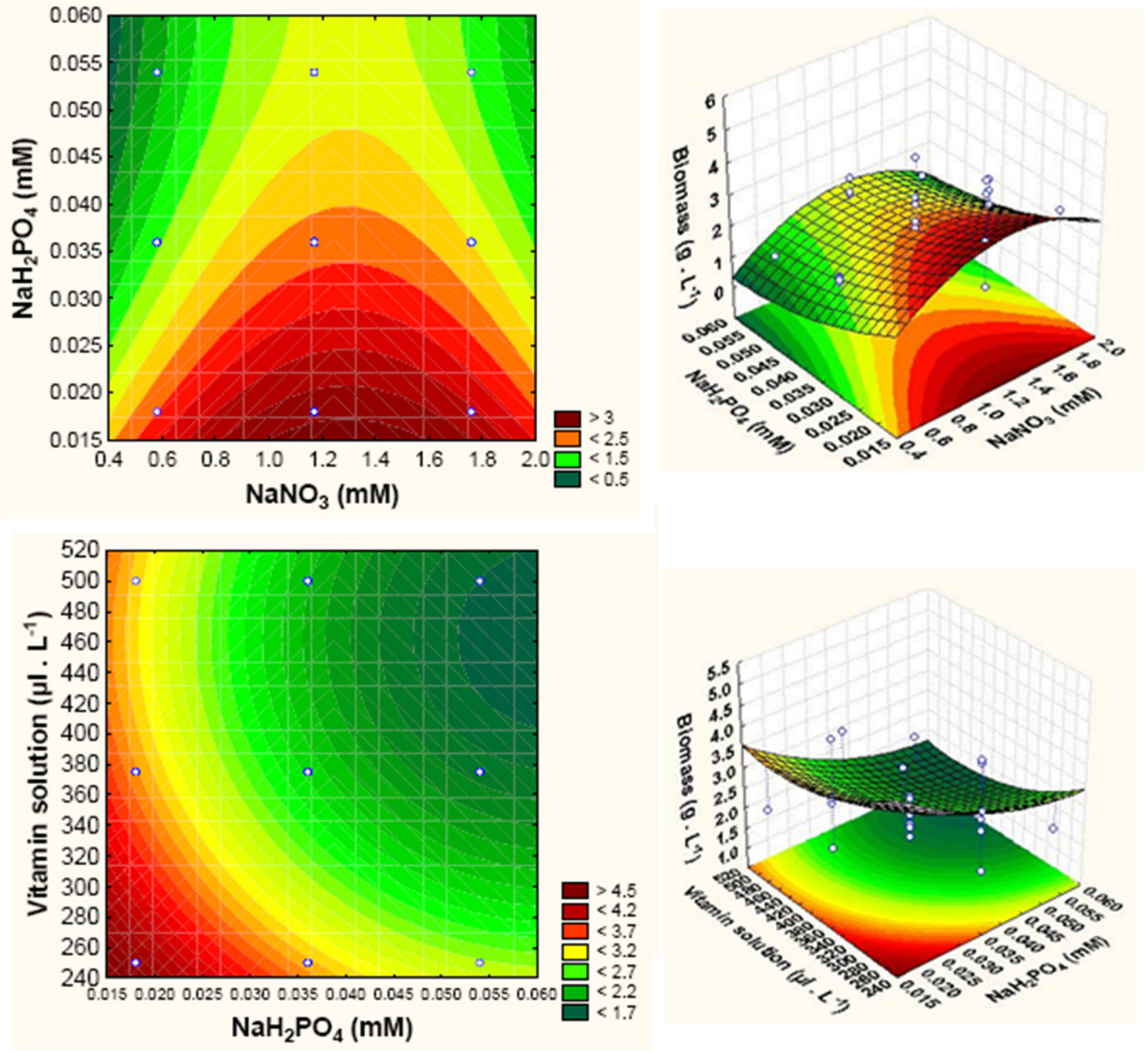
Contour plots and response surface plot showing the F/2 nutrients effect on biomass production

Figure 1 shows the 2D (contour plots) and 3D response curves allowing us to analyze the interaction of the four factors and their optimal levels on *Tetraselmis* sp. growth. The final biomass concentration (5.72 g.L^-1^) was obtained with 1.76 mM of NaNO_3_, 0.018 mM of NaH_2_PO_4_, 1500 μL.L^-1^ of metals solution and 312.5 μL.L^-1^ of vitamins solution in the culture medium.

#### Combined effects of modified-F/2 medium components on total chlorophylls and carotenoids productions

After identifying the significant variables for total chlorophylls (*Y*_2_) and carotenoids (*Y*_3_) production responses, the following second order response surface models were established, respectively, and illustrated in the equations (8) and (9):

8 $$ {\widehat{Y}}_2=\hbox{--} 9.48 + 2.78{x}_1\hbox{--} 0.92{x_1}^2 + 180.17{x}_2\hbox{--} 1378.60{x_2}^2 + 0.002{x}_3\hbox{--}\ 2.41\ 1{0}^{-6}{x_3}^2 + 0.02{x}_4\hbox{--}\ 3.19\ 1{0}^{-5}{x_4}^2\hbox{--}\ 20.24{x}_1{x}_2 + 3.1\ 1{0}^{-3}{x}_1{x}_4 + 5.04\ 1{0}^{-6}{x}_3{x}_4\left( P-\mathrm{value} < 0.05\right) $$

9 $$ {\widehat{Y}}_3=0.09\ {x_1}^2+2.66{x}_2\hbox{--}\ 72.01{x_2}^2+1.95{10}^{\hbox{-} 4}{x}_3\hbox{--} 1.28\ 1{0}^{-7}{x_3}^2 + 1.27\ 1{0}^{-3}{x}_4 + 3.06\ {x}_1{x}_2 + 5.561{0}^{-3}{x}_2{x}_3\left( P-\mathrm{value} < 0.05\right) $$

According to ANOVA results, the models corresponding to total chlorophylls (Table 5) and carotenoids (Table 6) contents responses revealed a high determination coefficients (R^2^ = 0.98 and R^2^ = 0.95, respectively) indicating the significance of the two models and the good agreement between predicted and experimental response results.

**Table 5:** Analysis of variance of the model for total chlorophylls production

Source	Sum of squares	df	Mean square	*F* value	*P* value
*x*_1_	6.45	1	6.45	1233.12	<0.05
*x*_2_	9.77	1	9.77	1866.88	<0.05
*x*_3_	0.13	1	0.13	26.49	<0.05
*x*_4_	8.45	1	8.45	1614.72	<0.05
*x*_1_ *x*_2_	0.18	1	0.18	35.33	<0.05
*x*_1_ *x*_3_	0.01	1	0.01	2.10	0.28
*x*_1_ *x*_4_	0.20	1	0.20	39.55	<0.05
*x*_2_ *x*_3_	0.04	1	0.04	8.42	0.10
*x*_2_ *x*_4_	0.001	1	0.001	0.30	0.63
*x*_3_ *x*_4_	0.39	1	0.39	75.84	<0.05
*x*_1_^2^	0.54	1	0.54	103.81	<0.05
*x*_2_^2^	1.06	1	1.06	203.32	<0.05
*x*_3_^2^	1.93	1	1.93	370.45	<0.05
*x*_4_^2^	1.32	1	1.32	252.65	<0.05
Lack of fit	0.63	10	0.06	12.04	0.07
Pure error	0.01	2	0.005		
Total	35.89	26			

R^2^ = 0 .98

**Table 6:** Analysis of variance of the model for carotenoids production

Source	Sum of squares	df	Mean square	*F* value	*P* value
*x*_1_	0.0008	1	0.0008	6.25	0.12
*x*_2_	0.02	1	0.02	188.48	<0.05
*x*_3_	0.003	1	0.003	25	<0.05
*x*_4_	0.014	1	0.014	105.06	<0.05
*x*_1_ *x*_2_	0.004	1	0.004	31.68	<0.05
*x*_1_ *x*_3_	0.0002	1	0.0002	1.68	0.32
*x*_1_ *x*_4_	0.0001	1	0.0001	0.75	0.47
*x*_2_ *x*_3_	0.01	1	0.01	75.00	<0.05
*x*_2_ *x*_4_	0.001	1	0.001	12.00	0.07
*x*_3_ *x*_4_	0.0006	1	0.0006	4.68	0.16
*x*_1_^2^	0.005	1	0.005	43.34	<0.05
*x*_2_^2^	0.002	1	0.002	21.77	<0.05
*x*_3_^2^	0.005	1	0.005	41.17	<0.05
*x*_4_^2^	0.0003	1	0.0003	2.77	0.23
Lack of fit	0.005	10	0.0005	3.89	0.22
Pure error	0.0002	2	0.0001		
Total	0.10	26			

R^2^ = 0.95

**Fig. 2:**
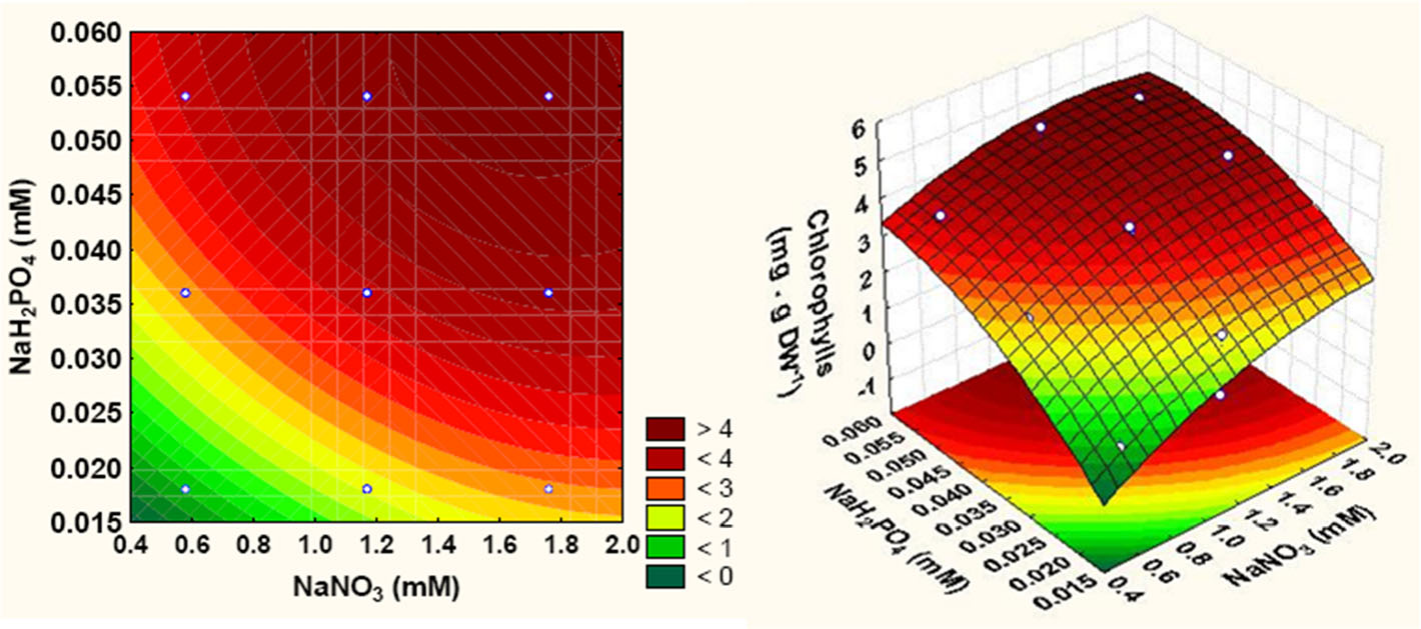
Contour plots and response surface plot showing the F/2 nutrients effect on total chlorophylls production

It was shown that the total chlorophylls production was influenced by all tested factors and their quadratic and interactions effects (Eq. 8). The interaction between independent factors affecting the total chlorophylls production can be estimated using the surface plot shown in Fig. 2.

In fact, increasing the extracellular NaNO_3_ and NaH_2_PO_4_ concentrations from 0.58 mM to 1.76 mM and from 0.018 mM to 0.054 mM, respectively, and rising the extracellular metals (1250 µL.L^-1^) and vitamins (500 µL.L^-1^) concentrations, the total chlorophylls production reached its maximum value of 5.18 mg.g DW^-1^.

The linear (except for NaNO_3_) and quadratic (except for vitamins) effects *P*-values indicated that all factors show great influence on carotenoids synthesis.

**Fig. 3:**
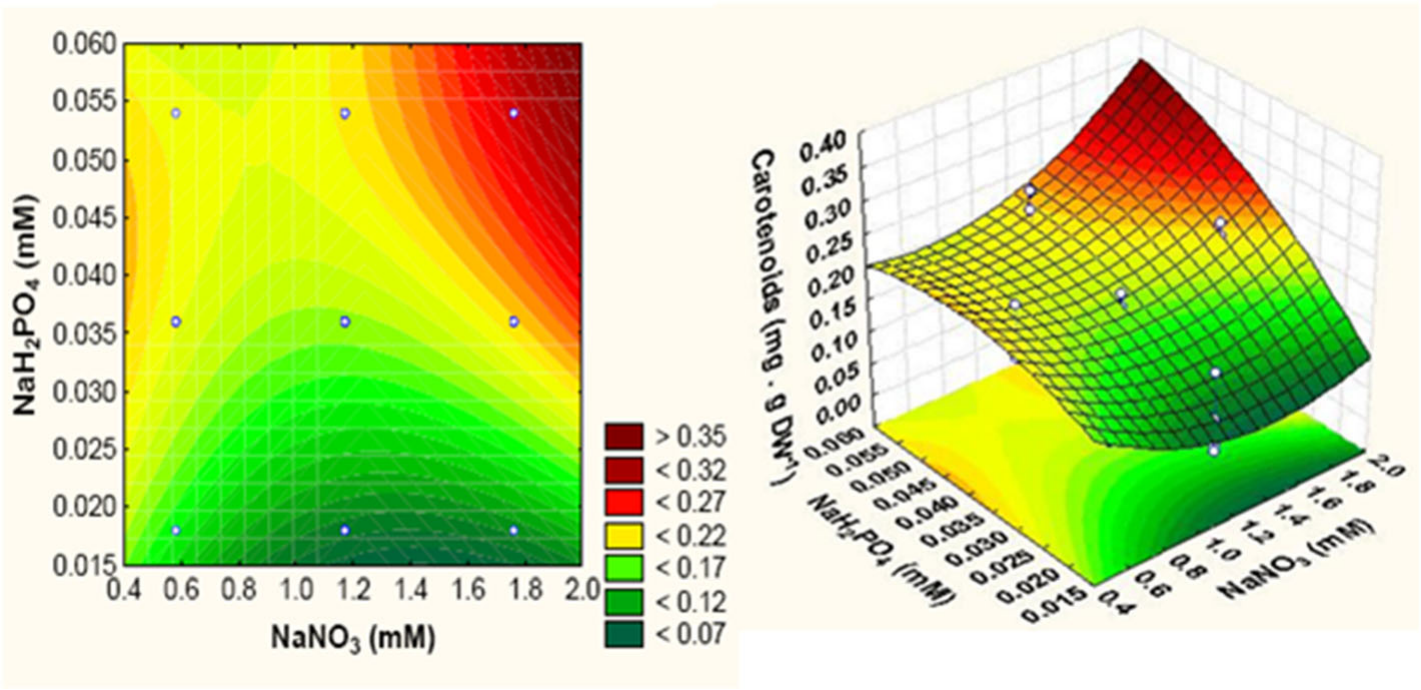
Contour plots and response surface plot showing the F/2 nutrients effect on carotenoids production

From 3D response surface plots and corresponding contour plots (Fig. 3), the maximum values of carotenoids production (0.3 mg.g DW^-1^) response was observed at the high level (+1 level) of all the factors.

#### Combined effects of modified-F/2 medium components on starch production

In this study, the second-order regression equation for starch production (*Y*_4_) was estimated through the following equation (Eq. 10):

10 $$ {\widehat{Y}}_4=\hbox{--}\ 0.40{x}_1+7.30{x}_1{x}_2 + 8\ 1{0}^{-4}{x}_1{x}_4\left( P-\mathrm{value}<0.05\right) $$

As seen in Table 7, the determination coefficient (R^2^) of 0.88 highlighted the significance of the model. However, the linear significant effect of nitrate (*x*_1_) (*P*-value < 0.05) was shown to be negative.

**Table 7:** Analysis of variance of the model for starch production

Source	Sum of squares	df	Mean square	*F* value	*P* value
*x*_1_	0.021	1	0.021	29.76	<0.05
*x*_2_	0.0006	1	0.0006	0.96	0.43
*x*_3_	0.0027	1	0.0027	3.85	0.19
*x*_4_	0.0006	1	0.0006	0.96	0.43
*x*_1_ *x*_2_	0.0240	1	0.0240	34.32	<0.05
*x*_1_ *x*_3_	0.0001	1	0.0001	0.14	0.74
*x*_1_ *x*_4_	0.0132	1	0.0132	18.89	<0.05
*x*_2_ *x*_3_	0.0025	1	0.0025	3.57	0.19
*x*_2_ *x*_4_	0.0049	1	0.0049	7.00	0.11
*x*_3_ *x*_4_	0.0036	1	0.0036	5.14	0.15
*x*_1_^2^	0.005	1	0.005	7.24	0.11
*x*_2_^2^	0.004	1	0.004	6.66	0.12
*x*_3_^2^	0.000004	1	0.000004	0.005	0.95
*x*_4_^2^	0.00002	1	0.00002	0.03	0.87
Lack of Fit	0.0095	10	0.00095	1.35	0.50
Pure error	0.0014	2	0.0007		
Total	0.093	26			

R^2^ = 0.88

**Fig. 4:**
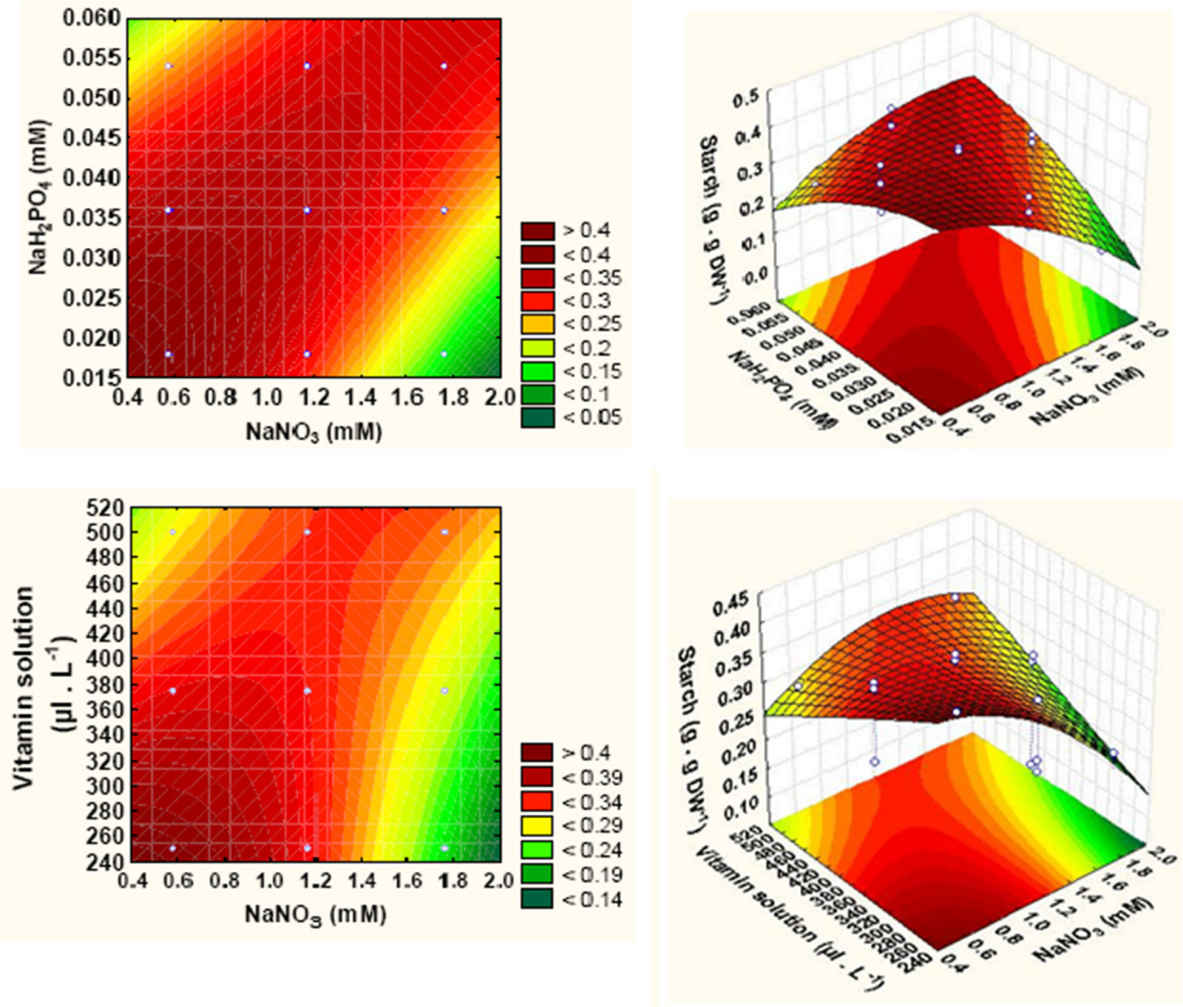
Contour plots and response surface plot showing the F/2 nutrients effect on starch production

The lower nitrate concentration coincides with the higher starch production in the tested experimental domain. A positive significant interaction (*P*-value < 0.05) was observed between nitrate and phosphate (*x*_1_ *x*_2_) and between nitrate and vitamins (*x*_1_ *x*_4_) (Eq. 10). The effect of phosphate, metals and vitamins was shown to be non significant for starch production (*P*-value > 0.05). The negative significant effect of nitrate (*x*_1_) seen in Eq. (10) indicated the antagonistic effect on starch content. The response observed in three dimension (3D) response surface and corresponding contour plots (2D) (Fig. 4) show the interactive effect of nutrients on starch production of *Tetraselmis* sp. According to the results shown in Fig. 4, the maximum of starch content value (0.42 g.g DW^-1^) was obtained when the concentrations of NaNO_3_ and NaH_2_PO_4_ decreased from 1.76 mM to 0.58 mM and from 0.054 mM to 0.027 mM, respectively, and when the volume of metal and vitamin solutions decreased from 1500 μL.L^-1^ to 1000 μL.L^-1^ and from 500 μL.L^-1^ to 250 μL.L^-1^, respectively. These results suggest that the highest starch content in *Tetraselmis* sp. strain V_2_ was obtained under nitrogen, phosphorus, metals and vitamins limitations.

### Carbohydrates detection by FT-IR spectroscopy

**Fig. 5:**
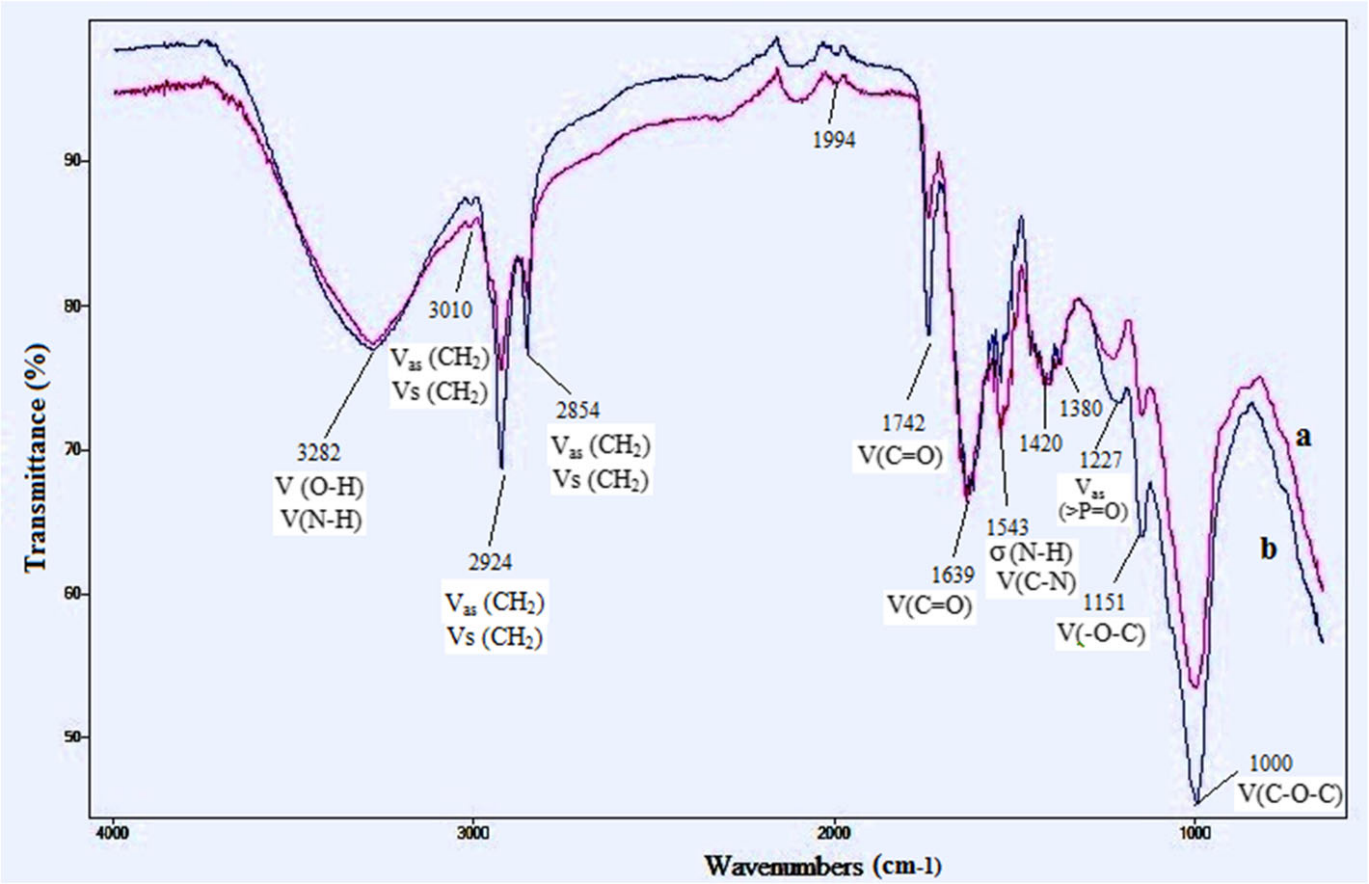
FT-IR spectra for *Tetraselmis* sp. (**a**) *Tetraselmis* sp. cultivated under standard medium culture (factors at zero levels), (**b**) *Tetraselmis* sp. cultivated under optimized medium culture for starch production

FT-IR spectra of *Tetraselmis* sp*.* cells showed twelve distinct bands over the wavenumber ranging from 4000 to 600 cm^-1^ (Fig. 5). These absorption bands were attributed to specific molecular groups based on published data [18]. In this study, the spectrum of proteins which peak is at 1639 cm^-1^ (between 1590 and 1650 cm^-1^) corresponds to the N-H and C = O of amides I, whereas the peak at 1534 cm^-1^ (between 1500 and 1560 cm^-1^) shows the presence of the N-H groups and asymmetric N = O groups of amide II. Moreover, these infra red spectra show the presence of an important peak at 3282 cm^-1^ allocated to the vibrations of the stretchings O-H groups and N-H groups of proteins.

The spectrum of the lipids was characterized by characteristic bands at 1420 cm^-1^ and 1380 cm^-1^ which were due mainly to the C-O-H stretching vibrations of the carboxylic acids groups of the lipids. In addition, the peak at 1742 cm^-1^ was associated with lipids and fatty acids which are primarily due to V (C = O) of ester groups. Finally, the successive bands at 1000, 1151 and 1227 cm^-1^ show the stretching of the bands of O-C groups of carboxylic acids. The carbohydrates features are characterized by two bands, at 1151 and 1000 cm^-1^, assigned to V (−O-C) stretching vibrations of the pyranosic cycles of the polysaccharides. The nucleic acids have functional groups at 1227 cm^-1^ associated with V_as_ (>P = O) stretching attributed to phosphorus compounds such as the phosphodiesters. The absorption bands at 1380 and 1420 cm^-1^ prove the presence of sulfates groups (S = O).

In this comparative study, the FT-IR analysis showed no significant changes in the qualitative biochemical composition of *Tetraselmis* sp. cultivated under optimized medium culture for starch production compared to the standard medium culture (factors at zero level).

## Discussion

Recently, several studies, focused on research renewable sources of energy, have proved that microalgae present an important source for biofuel production [19, 20]. Thus, the optimizations of biomass productivity, lipids and carbohydrates productions are in fact an important approach for a more economical biofuel production. The availability of nutrients such as phosphorus, nitrogen, metals, and vitamins is one of the most important factors regulating cells growth, photosynthesis and other processes in microalgae.

Nitrogen and phosphorus were the two main components that play an essential role and a significant effect on the growth of *Tetraselmis* sp. strain V_2_ (Fig. 1 and Table 4). The present study showed that the highest biomass concentration (5.72 g.L^-1^) and biomass productivity (408.57 mg.L^-1^.day^-1^) were obtained under high metals and nitrogen concentrations (1.76 mM) and limited vitamins and phosphorus concentrations (0.018 mM). Therefore, the biomass productivity increased by approximately 2.4-fold at optimized conditions compared to standard ones.

Similar results, found by Yao et al. [2], proved that with 11 mM of KNO_3_ in the culture medium of *Tetraselmis subcordiformis* led to the higher biomass concentration (5.72 g.L^-1^) after 8 days of growth. Our findings proved that *Tetraselmis* cells growth was inhibited upon nitrogen starvation (Fig. 1).

Our results satisfied the hypothesis of Xin et al. [21] saying that with the increase of extracellular phosphate concentration, the biomass of microalgae decreased. In another study, the authors showed that the high concentration of phosphate decreased the growth of C*hlorella* [22].

In this study, Metals (FeCl_3_,6H_2_O; Na_2_EDTA,2H_2_O; CuSO_4_,5H_2_O; Na_2_MoO_4_,2H_2_O; ZnSO_4_,7H_2_O; CoCl_2_,6H_2_O; MnCl_2_,4H_2_O) presented a quadratic positive significant effect on cells growth. Our results showed that the highest biomass productivity (408.57 mg.L^-1^.day^-1^) was achieved with 0.015 mM iron concentration (data not shown). Sun et al. [23] noted that the biomass productivity of *N. oleobundans* HK-129 increased to 292.83 mg.L^-1^.day^-1^ with the increase of Fe^3+^ concentration to 0.037 mM, which is in agreement with the present findings.

In photoautotrophic photosynthesis, iron (Fe^3+^) was an essential cofactor for photosystems I and II activities. Therefore, iron affects the microalgae growth by affecting light harvesting, electron transfer, energy conversion and carbon fixation [24]. Iron limitation can also decrease the photosynthesis efficiency and biomass concentration [23].

The measurement of pigments such as total chlorophylls and carotenoids was established to analyze the effect of nutrients of F/2 medium and their interactions on photosynthetic pigments production in *Tetraselmis* sp. (Tables 5 and 6). The highest total chlorophylls (5.18 mg.g DW^-1^) and carotenoids (0.3 mg.g DW^-1^) contents were obtained under high extracellular concentration of all F/2 nutrients (Figs. 2 and 3). Just like the current study, Yao et al. [2] found that the maximal photosystem II activity F_*v*_/F_*m*_ increased with the increase of KNO_3_. Thus, chlorophylls content attained 4.9% at day 2 and at 11 mM of KNO_3_, while it decreased to 1.5% DW at day 8 when KNO_3_ was exhausted. In another study, the maximum of total chlorophylls and carotenoids productions were found in *A. falcatus* cultured in BBM medium [25] which contains a high nitrate and phosphate concentrations.

Relying on the nutrients optimization, our data showed that the maximum of starch content of 42% DW was obtained at low nitrogen, phosphorus, metals and vitamins concentrations in the culture medium of *Tetraselmis* sp. Therefore, the starch content increased by 1.3-fold in the optimized F/2 medium culture compared to that at zero level (standard conditions).

The findings obtained in our research are interesting when compared to previous studies; see the findings summarized in Table 8.

**Table 8:** Starch production in different microalgae under different culture conditions, as reported in the literature

Microalgae	Stress conditions	Starch content (% of DW)	References
*T. subcordiformis*	–P (low ICD)	44.1	[6]
*T. subcordiformis*	–P (medium ICD)	42.2	[6]
*T. subcordiformis*	–N	54.0	[2]
*Chlorella vulgaris*	–N	41	[5]
*Chlorella vulgaris Beijerinck CCALA924*	–N	37	[7]
*Chlorella vulgaris Beijerinck CCALA924*	–P	53	[7]
*Tetraselmis* sp.	Optimized F/2 medium	42.3	This study

A similar phenomenon has been observed in other microalgae [7, 8, 26–28]. Yao et al. [6] reported also that starch production in *T. subcordiformis* increased to a maximum of 44.1% under phosphorus limiting conditions and low initial cell densities (ICD).

It was also reported that *Chlorella vulgaris* produced 41% DW of starch under nitrogen starvation [5]. It was already suggested that phosphorus deprivation induced starch accumulation (53%) in *Chlorella vulgaris Beijerinck* CCALA924 [7]. As shown in Table 3, total chlorophylls content is inversely proportional to that of starch. It can be observed that at high phosphorus concentration (Runs 26 and 4) and high nitrogen concentration (Runs 13 and 22), the total chlorophylls content was high. Inversely, the Runs 1 (low NaNO_3_ and NaH_2_PO_4_ concentrations), 10 and 19 (low NaNO_3_ concentration) and 16 (low NaH_2_PO_4_ concentration) gave a high starch content, while the chlorophylls, which are compounds with high nitrogen content, were very low. The third and fourth parameters were low (between negative level (−1) and zero level (0)). Similarly, Yao et al. [2] pointed out that the maximal photosystem II activity (*F*_*v*_ */F*_*m*_) increased at high extracellular KNO_3_ concentration corresponding to a decrease of starch content, which is in agreement with our results and those of [29] with *Chlorella vulgaris*.

The results described in our research are similar to those described by [28] suggesting that the phosphorus deprivation can lead to the deflection of metabolism from growth and photosynthesis to energy compounds such as lipids and carbohydrates. The present work clearly reveals that the decrease of initial nitrogen and phosphorus concentrations as well as metals and vitamins elements in *Tetraselmis* sp. strain V_2_ culture resulted on a maximum of starch production (Fig. 4). However, the chlorophylls content increased with the increase of nitrogen and phosphorus source in *Tetraselmis* sp. These results are similar to those achieved by [6]. Thus, nitrogen and phosphorus limitations caused the restriction of photosynthesis, protein and chlorophylls synthesis and the accumulation of carbohydrates and lipids [8, 25, 30]. In addition, when the nitrogen and phosphorus concentrations increased, the flow of photosynthetic carbon was diverted into chlorophylls and protein synthesis [2]. Brányiková et al. [7] assumed that the addition of cycloheximide in the medium led to the inhibition of protein synthesis and increase the starch content to below 60% DW in *Chlorella vulgaris.* Therefore, the nutrient stress condition such as the lack of nitrogen and phosphorus might redirect the metabolism from chlorophylls and proteins to starch accumulation. Similar results were shown with *Tetraselmis subcordiformis* [2], *Chlamydomonas reinhardtii* [31] and *Dunaliella salina* [32].

Furbank and Lilley [33] demonstrated that the high free phosphate concentration accumulated in the chloroplast and cytosol was toxic to the metabolism of microalgal cells, particularly to their photosynthesis [34]. In addition, it was known that starch synthesis in many plants and microalgae was carried out by the conversion of ATP and glucose-1-phosphate to pyrophosphate and ADP-glucose. This reaction was catalyzed by ADP-glucose pyrophosphorylase (AGPase) [35]. This enzyme was inhibited by free orthophosphate (Pi) and it was activated by the 3-phosphoglyceric acid (3-PGA) molecule. Therefore, the 3-PGA/Pi ratio regulated the AGPase activity [36]. The increase of the photosynthetic activity facilitated 3-PGA production with a higher 3-PGA/Pi ratio essential for the activation of AGPase and thus made the starch synthesis more efficient [6]. This observation could explain the phenomenon of the current study showing that the lower phosphorus concentration increased the starch production. In this study, however, the metals concentration does not have a significant effect on starch synthesis in *Tetraselmis* sp. The maximum of starch content was obtained at zero level of metals solution volume. These findings confirm some previous results of [5], which demonstrated that irons concentration did not affect starch production in *C. vulgaris*. Here, the metals solution contained FeCl_3_,6H2O; Na_2_EDTA,2H_2_O; Na_2_MoO_4_,2H_2_O; ZnSO_4_,7H_2_O; CoCl_2_,6H_2_O; MnCl_2_,4H_2_O and CuSO_4_,5H_2_O which sulfur concentration was very low (12.10^-5^ mM) in the optimized medium culture for a maximal starch production. This result is similar to that of [2] which obtained a maximum of starch content of 62.1% DW in *T. subcordiformis* at 0 mM of MgSO_4_. In fact, the low sulfur concentration caused a decrease of the photosystem II activity. Therefore, the microalgal cells were exposed to culture stress conditions, hence increasing starch synthesis. In this study, Run 16 (Table 3) showed a high biomass concentration of 4.8 g.L^-1^ with a high biomass productivity of 343 mg.L^-1^.day^-1^ (data not shown) corresponding to a high starch content of 36% DW. Thus, this result is crucial to achieve a compromise between increasing cell growth and starch accumulation in *Tetraselmis* sp. strain V_2_ in order to set up a promising source for bioethanol production. Based on the FT-IR spectra analysis, the relative carbohydrate content was calculated by measuring the ratio of the transmittance areas of the carbohydrate bands (1200–950 cm^-1^) to the amide I band (1639 cm^-1^). Thus, the carbohydrates/amide I ratio showed an increase of 16% in *Tetraselmis* sp. cultivated under optimized medium culture for starch production than the standard medium culture (Table 1), increasing from 7.15 to 8.29 of carbohydrates/amide I ratio. Here, the carbohydrates/amide I ratio of 8.29 for *Tetraselmis* sp. cultivated under optimized medium culture was higher than that obtained by *T. subcordiformis* and *Chaetoceros* sp. (3.07 and 2.30, respectively) [19].

As shown in this study and in previous ones, nitrogen and phosphorus limitation induced starch biosynthesis, as noted by high carbohydrates/amide I ratio [19].

## Conclusions

In summary, marine microalga *Tetraselmis* sp. V_2_ reached 5.72 g.L^-1^ and 408.57 mg.L^-1^.day^-1^, respectively, at high metals and nitrogen concentrations and low vitamins and phosphorus ones. This was a 2.4-fold higher increase than that obtained at the standard conditions. Our study also shows that the maximum of starch content of 42% DW was obtained under nitrogen, phosphorus, metals and vitamins limitations, while the highest total chlorophylls content of 5.18 mg.g DW^-1^ and carotenoids content of 0.3 mg.g DW^-1^ were achieved at high nitrogen, phosphorus, metals and vitamins concentrations. Therefore, the isolated microalgae *Tetraselmis* sp. strain V_2_ has a good potential for biomass and starch production considering its high growth and starch content, which makes this strain a potential feedstock for bioethanol production.
